# Slabs from the Furnace

**Published:** 1860-10

**Authors:** Joe Stackpole


### SLABS FROM THE FURNACE.

BY JOE STACKPOLE.

Much has been said and written upon the subject of practical dentistry, and much remains to be said to aid the inquiring student in the dreary toil that he is necessarily subjected to when he first assumes the responsibility of those most valuable behests which he desires to preserve from the ravages of disease.

The abstract principle of dental phenomena, and dental manipulation, has been ground through a thousand mills, and left the public and the profession little benefited.

Oceans of ink, and warehouses full of paper, have been exhausted to illustrate a few plain truths, which if narrated in a simple, practical manner, would have saved the student the trouble of wading through folio after folio, too ambiguous for the young, and too profound for the more advanced.

There seems to be a difficulty, in these days of locomotive and telegraphic communications, in simplifying; or in other words, of resorting to the hand-barrow and cart to convey our goods to market.

Some new terms must be sought—some mystic word must be paraded through every article in order that genius be made apparent in the profusion of words and symbols.

It has been wisely said that much dignity, with corresponding taciturnity, make many little men great. So it may be remarked of many of our would be great dentists. They are gifted in words and technicals, rather than in true practical knowledge. Hence the solution of a fact generally conceded, that great dental theorists are indifferent, if not absolute ignoramuses in the practical detail; and it also illustrates another fact, viz: that it is much easier for some men *to preach than to practice.*

Scientific men have done much to exhume the dental profession from a state of ignorance, scarcely paralleled by any other trade or calling. But all theory fails when it results in no benefit to the public. It is an easy matter to theorize upon the practicability of flying through the air with the rapidity of a bird on wing, or of the great utility of a perpetual motion, and it appears to many perfectly feasible; but of what benefit are these speculations to mankind?

Newton, Franklin, Fulton, and a thousand others of equal note, each in their day, were consummate theorists, and each in turn were saddled with the opprobrium of visionary. But the world has been no less astonished than benefited, by practical results drawn from the fountain of knowledge.

The practical Hercules in the dental profession, is the great philanthropist of the age. The young lean upon him with confiding trust, for their present and future health, happiness, and for personal charms. The aged are indebted to him for much of the comfort that remains to them.

The dental profession is born in practice more than any other, and survives only by practice. It admits of no relaxation. The practiced hand becomes a stranger to its art by diversion.

A grand flourish of technicals, with the addition of a borrowed title, as disgraceful to the claimer, as to the august body which gave it, has consigned many poor victims to a life of wretchedness, known only to those whose comfort and brightest hopes have been crushed by the mal-practice of these inhuman monsters.

In illustration I will cite a case, that can be referred to when necessary.

Miss M——, daughter of Lieut. M——, of the U. S. A., was placed in the hands of a dentist in this city, to have her front teeth filled. He filled them several times in the course of three years, but the fillings would not "stick," and at the end of that time he was obliged to paralyze the nerves of four of her front teeth. He filled them several times subsequently with no better success; finally he became tired of these gratuitous operations, and refused to attempt further to save them by filling with gold, but promised her if she would call once a month, that he would fill them with *white wax.* The teeth are now permanently filled, though their beauty is destroyed.

What fond anticipations have been crushed by this monster of a dentist! Who can imagine the feelings of a parent at a loss so overwhelming, and so irreparable? And yet his *honorably* obtained, high sounding title, with a little extra flourish of his dental lore, satisfies his friends, silences his enemies, and shields him from a just retribution; and on he goes to the next victim to his audacity.

Such scenes are of almost daily occurrence, and yet the humbugged public swallow the catastrophe as though it was a mere joke.

Neither position, nor honorable title, is satisfactory evidence of profound knowledge, or of consummate skill, in the dental profession. It may be asked why such villainy is not exposed, and the public thereby protected, by those who are acquainted with these facts, and whose duty it is to do it. The answer is plain. Kings have been assassinated by miscreants whose only object was public notoriety. So these impostors and charlatans challenge exposure, for the notoriety and the benefit of crying persecutions.

The fault of imposition and empiricism is not alone confined to the profession. The tendency of the public to *imbibe* every new theory upon the mere *say so* of some visionary dreamer, is proverbial. Change is sought after in Church, as well as in State, in politics as well as in religion ; and however absurd the proposition, the credulous public are always ready to embrace it. The Churches, in their multitudinous tenets, are the promulgators of more ridiculous humbugs, perhaps, than any other portion of the community. The clergy usually take the first pill, probably at a discount, and if the discovery is made by a good brother in *our* particular faith, the effect is most salutary. From this respectable head, the influence descends to the vestry, and from thence to the laity, and so on down to the Church sewing society ; and here, in fact, is the fulcrum that moves the whole popular current.

All subjects are here freely discussed, excepting that most appropriate to the occasion. Matrimony is usually the leading topic. From this stand-point the subject diverges into all sorts of ridiculous peradventure.

These Societies are the mouth-pieces of the Church. Not a kitchen or parlor escapes their surveillance. Without a shade of testimony, positive construction is put upon all acts, words, and thoughts, that do not chime in with this delectable clique. The seeds of slander, sown in a pious way, thro the operation of this humane institution, spring up in a thousand places, and in ten thousand forms; with an additional ten thousand constructions, carrying all before them into the labyrinth of doubt and mystery.

Physicians, Judges, and Lawyers are easily ensnared by this gullible influence. They are influenced to approve or condemn, without a solitary fact to base their decisions upon, other than brother Zebedee's information. Letters of introduction or recommendation are obtained to establish confidence, and to secure the patronage of most valued and esteemed friends ; and this, too, by impostors, whose ignorance is only known by acquaintance, and whose villainy is only appreciated by contact. These monsters have no habitation. Their very subsistence depends upon the credulity and gullibility of strangers. They migrate from place to place, carrying the confidence of friend to friend, in order to promote individual interest through the basest empiricism, and at the greatest personal sacrifice.

The people judge honestly of the whole profession in consequence of the numerous victims to this nefarious practice. But this mode of establishing confidence for stipend, is not confined to town or country. These *gentlemen* (Heaven save the mark!) inhabit cities, and here they find a rich harvest. Their sins are hidden, or covered through the instrumentality of Church vows and Church influence—through the aid and sympathy of social societies and fraternal relations, or through the power and efficacy of individual friendship. Truth, self-respect, and even the solemnity of an oath—all are profaned and sacrificed to the one paramount object, *self-interest;* or what is equally humiliating and degrading, self-aggrandizement. This is the sole motive power, to which everything holy or noble in the character of man must be made subservient.

The physician is called to the sick friend, with strong reliance in his skill and judgment. The friend dies, but he has the happy consolation of having done his duty. In case of failure, the dentist can find no such consolation. He is called to administer to organs over which judgment and skill can, under ordinary circumstances, triumph. These organs are as valuable to the prospects, hopes and happiness of his patient as life itself, for without them, all but life would be sacrificed. What, then, is his crime if he fails, and what should be his penalty?

In this exordium I do not wish to be considered censorious in any manner to the better informed on this subject. My object is to show the necessity of popular information, adapted to every capacity of intellect, in order that the evils may be corrected, that are now indiscriminately and unjustly charged upon the "just as well as the unjust," and that the public generally may understand the diagnosis of dental diseases and of dental manipulation sufficiently to protect their children and friends against a loss cruel and irretrievable. You may hear from me again.

